# Crystal structures of the synthetic inter­mediate 3-[(6-chloro-7*H*-purin-7-yl)meth­yl]cyclo­butan-1-one, and of two oxetanocin derivatives: 3-[(6-chloro-8,9-di­hydro-7*H*-purin-7-yl)meth­yl]cyclo­butan-1-ol and 3-[(6-chloro-9*H*-purin-9-yl)meth­yl]cyclo­butan-1-ol

**DOI:** 10.1107/S2056989019004432

**Published:** 2019-05-03

**Authors:** Ayat Yaseen, Muhammad Murtaza Hassan, Edward Lee-Ruff, Gerald F. Audette

**Affiliations:** a Department of Chemistry, York University, 4700 Keele St., Toronto, ON, M3J 1P3, Canada

**Keywords:** crystal structure, oxetonacin, cyclo­butanone, cyclo­butanol, HIV, hydrogen bonding, offset π–π inter­actions, supra­molecular framework

## Abstract

The crystal structures of an inter­mediate, C_10_H_9_ClN_4_O, 3-[(6-chloro-7*H*-purin-7-yl)meth­yl]cyclo­butan-1-one (I), and two N-7 and N-9 regioisomeric oxetanocin nucleoside analogs, C_10_H_13_ClN_4_O, 3-[(6-chloro-8,9-di­hydro-7*H*-purin-7-yl)meth­yl]cyclo­butan-1-ol (II) and C_10_H_11_ClN_4_O, 3-[(6-chloro-9*H*-purin-9-yl)meth­yl]cyclo­butan-1-ol (IV), are reported. The crystal structures of the nucleoside analogs confirmed the reduction of the N-7- and N-9-substituted cyclo­butano­nes with LiAl(OtBu)_3_ to occur with facial selectivity, yielding *cis*-nucleosides analogs similar to those found in nature. Reduction of the purine ring of the N-7 cyclo­butanone to a di­hydro­purine was observed for compound (II) but not for the purine ring of the N-9 cyclo­butanone on formation of compound (IV). In the crystal of (I), mol­ecules are linked by a weak Cl⋯O inter­action, forming a 2_1_ helix along [010]. The helices are linked by offset π–π inter­actions [inter­centroid distance = 3.498 (1) Å], forming layers parallel to (101). In the crystal of (II), mol­ecules are linked by pairs of O—H⋯N hydrogen bonds, forming inversion dimers with an *R* ^2^_2_(8) ring motif. The dimers are linked by O—H⋯N hydrogen bonds, forming chains along [001], which in turn are linked by C—H⋯π and offset π–π inter­actions [inter­centroid distance = 3.509 (1) Å], forming slabs parallel to the *ac* plane. In the crystal of (IV), mol­ecules are linked by O—H⋯N hydrogen bonds, forming chains along [101]. The chains are linked by C—H⋯N and C—H⋯O hydrogen bonds and C—H⋯π and offset π–π inter­actions [inter­centroid distance = 3.364 (1) Å], forming a supra­molecular framework.

## Abstract 2

Two oxetanocin A derivatives have been synthesized stereospecifically. The crystal structures of both derivatives and a synthetic inter­mediate are reported on herein.

## Chemical context   

Derivatives of naturally occurring nucleotides are an emerging class of anti­viral therapeutics that are used to target tumors, herpes virus and the human immunodeficiency virus (HIV) (De Clercq, 2005 ▸). The development of new and different nucleoside analogs is important in combating drug-resistant mutants and increasing therapeutic effectivity. The naturally occurring oxetanocin A, a nucleoside analog, demonstrated efficacy against herpes and HIV (Hoshino *et al.*, 1987 ▸). Further exploration of oxetanocin A derivatives such as cyclo­but-A and cyclo­but-B (Lobucavir) represented an increase in potency and metabolic stability (Hoshino *et al.*, 1987 ▸; Bisacchi *et al.*, 1991 ▸). The current study focuses on the structural characterization of two nucleoside analogs, (II) and (IV), as well as the purinyl-cyclo­butanone inter­mediate (I), prior to reduction.
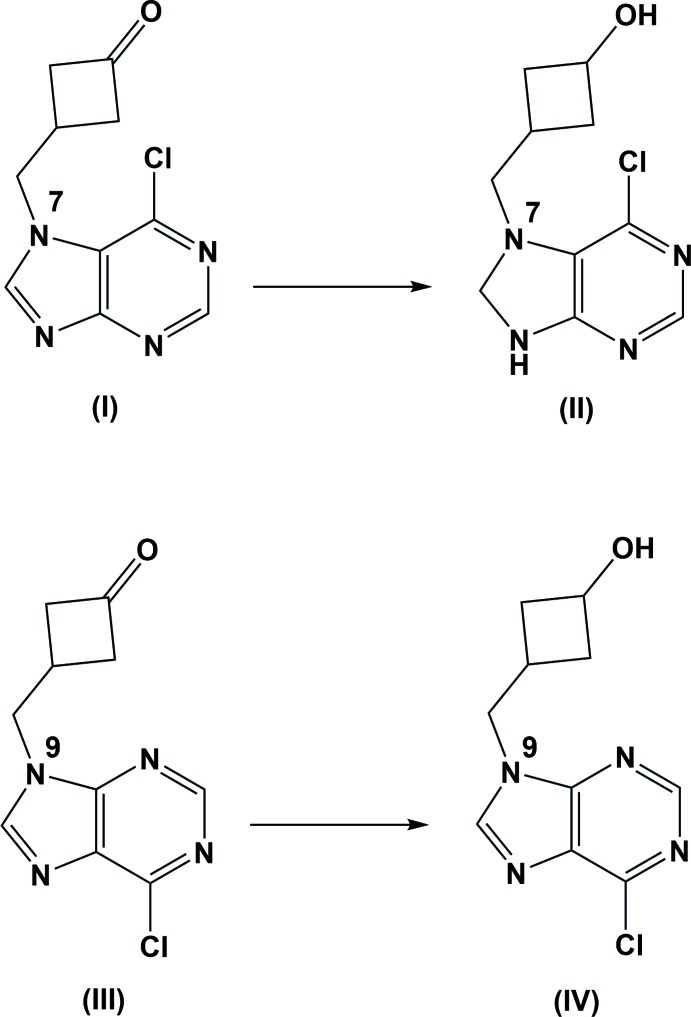


## Structural commentary   

**Figure 1:**
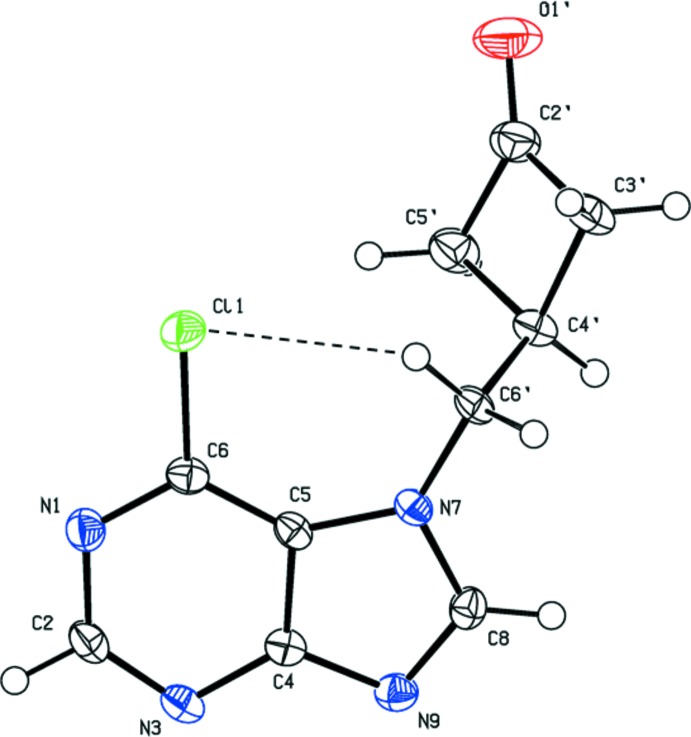
The mol­ecular structure of compound (I), with the atom labelling. Displacement ellipsoids are drawn at the 50% probability level. The intra­molecular C—H⋯Cl inter­action (Table 1 ▸) is shown as a thin dashed line.

The mol­ecular structures of compounds (I), (II) and (IV) are illustrated in Figs. 1 ▸, 2 ▸ and 3 ▸, respectively. In compounds (I) and (II) there is a short intra­molecular C—H⋯Cl inter­action present (Tables 1 ▸ and 2 ▸, respectively).

In compound (I) the purine ring is attached to the cyclo­butanone unit through atom N7, rendering the attachment *cis* to the chlorine atom bound to the aromatic ring at the C6 position. The mean plane of the cyclo­butane ring (*A* = C2′–C5′) is inclined to the mean plane of the purine ring system (*B* = N1/N37N7/N9/C2/C4/C5/C6/C8) by 52.62 (11)°, while the torsion angle N7—C5′—C4′⋯C2′ is *ca* 125.4°.

**Figure 2:**
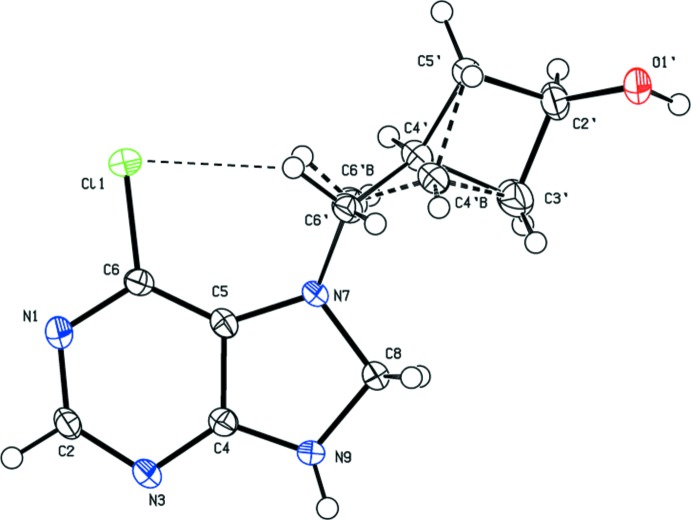
The mol­ecular structure of compound (II), with the atom labelling. Displacement ellipsoids are drawn at the 50% probability level. The intra­molecular C—H⋯Cl inter­action (Table 2 ▸) is shown as a thin dashed line. The minor fraction of the disordered atoms C4′ and C6′, *i.e*. C4′*B* and 6′*B*, are shown with dashed bonds.

Reduction of compound (I) with lithium tri-*tert*-but­oxy­aluminum hydride lead to the formation of the oxetanocin derivative compound (II). Here the the mean plane of the cyclo­butanol ring (*A*) is inclined to the mean plane of the purine ring system (*B*) by 26.37 (15)°, while the torsion angle N7—C5′—C4′⋯C2′ is *ca* 120.0°. Atoms C6′ and C4′ are positionally disordered and were split giving a refined occupancy ratio for C6′:C6′*B* and C4′:C4′*B* of 0.858 (4):0.142 (4) (Fig. 2 ▸).

**Figure 3:**
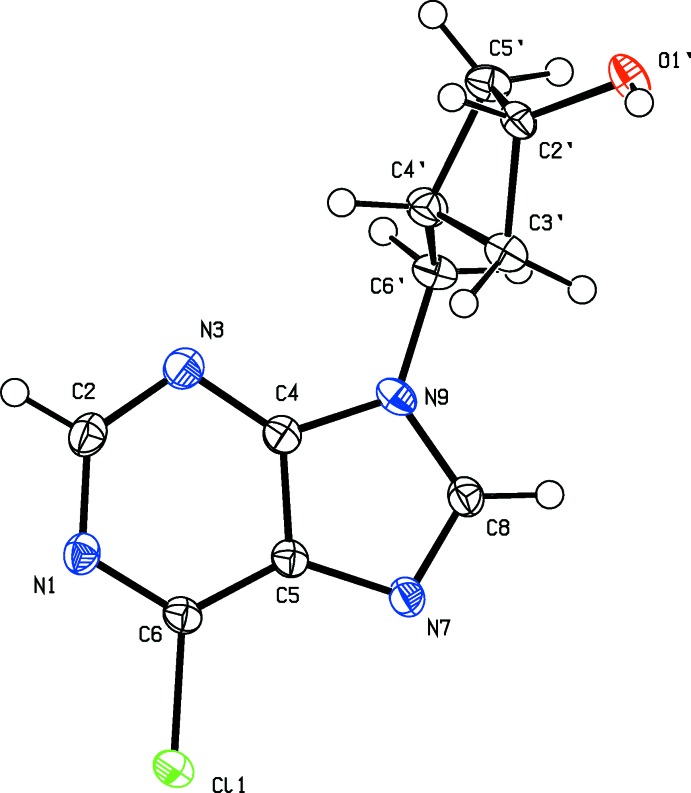
The mol­ecular structure of compound (IV), with the atom labelling. Displacement ellipsoids are drawn at the 50% probability level.

In compound (IV), the cyclo­butanol ring is attached to atom N9 of the purine ring (Fig. 3 ▸). As a result of the *trans* positioning of the cyclo­butanol unit, there are no intra­molecular hydrogen bonds between the chlorine atom and the cyclo­butanol or methyl­ene connector as observed in compounds (I) and (II). Here, the mean plane of the cyclo­butanol ring (*A*) is inclined to the mean plane of the purine ring system (*B*) by 71.20 (13)°, and the torsion angle N7—C5′—C4′⋯C2′ is *ca* 144.8°.

Reduction of the purine ring of the N-7 cyclo­butanone to a di­hydro­purine was observed for compound (II) but not for the purine ring of the N-9 cyclo­butanone on formation of compound (IV). This is confirmed by the values of the bond lengths and bond angles involving atom C8; see Table 3 ▸. Similar over-reductions of purine derivatives can be found in the literature, where electron-deficient purines are dearomatized by NaBH_4_ to a di­hydro­purine (Aarhus *et al.*, 2014 ▸). We speculate the reason for over-reduction of the N-7 ketone may be due to the strain associated with the system. N-7 alkyl­ation forces the chlorine of the purine ring to be oriented towards the cyclo­butanone ring, which increases the strain energy of the system. This strain energy is released when the rigid aromatic structure of the purine is reduced to a more flexible di­hydro­purine (*sp* ^2^ C8 to *sp* ^3^ C8). This strained orientation is not observed for the N-9 ketone, hence the integrity of its purine ring is preserved.

## Supra­molecular features   

**Figure 4:**
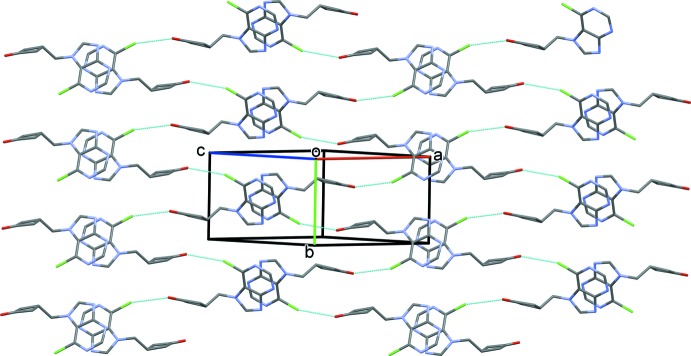
Crystal packing of compound (I), viewed normal to (101). The weak inter­molecular Cl⋯O inter­actions are shown as dashed lines. For clarity, the C-bound H atoms have been omitted.

In the crystal of (I), mol­ecules are linked by a weak Cl⋯O inter­action [Cl1⋯O1′(−*x* + 1, *y* − 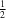, −*z* + 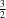) = 3.180 (2) Å], forming a 2_1_ helix along [010], see Fig. 4 ▸. The helices are linked by offset π-π- inter­actions, forming layers parallel to (101): *CgB*⋯*CgB* ^i^ = 3.498 (1) Å, *CgB* is the centroid of the purine ring system, α = 0.00 (5) Å, β = 21.6°, inter­planar distance = 3.252 (1) Å, offset = 1.289 Å, symmetry code (i) −*x* + 2, −*y* + 1, −*z* + 1.

**Figure 5:**
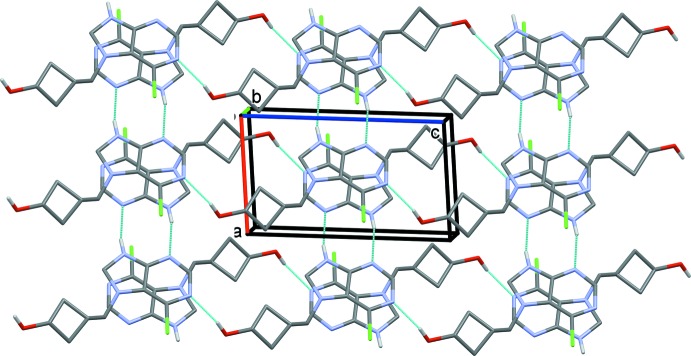
Crystal packing of compound (II), viewed along the *b* axis. The N—H⋯N and O—H⋯N hydrogen bonds (Table 2 ▸) are shown as dashed lines. For clarity, the C-bound H atoms have been omitted. The minor components of the disordered atoms C4′ and C6′ (*i.e*. C4′*B* and 6′*B*) have been omitted.

In the crystal of (II), mol­ecules are linked by pairs of N—H⋯N hydrogen bonds, forming inversion dimers with an 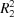
(8) ring motif (Table 2 ▸ and Fig. 5 ▸). The dimers are linked by O—H⋯N hydrogen bonds, forming ribbons along [001], which in turn are linked by C—H⋯π (Table 2 ▸) and offset π–π inter­actions, forming slabs parallel to the *ac* plane. [Details of the offset π–π inter­actions: *CgB*⋯*CgB* ^v^ = 3.498 (1) Å, *CgB* is the centroid of the purine ring system, α = 0.00 (5) Å, β = 21.6°, inter­planar distance = 3.252 (1) Å, offset = 1.289 Å, symmetry code (v) −*x* + 2, −*y* + 1, −*z* + 1.]

**Figure 6:**
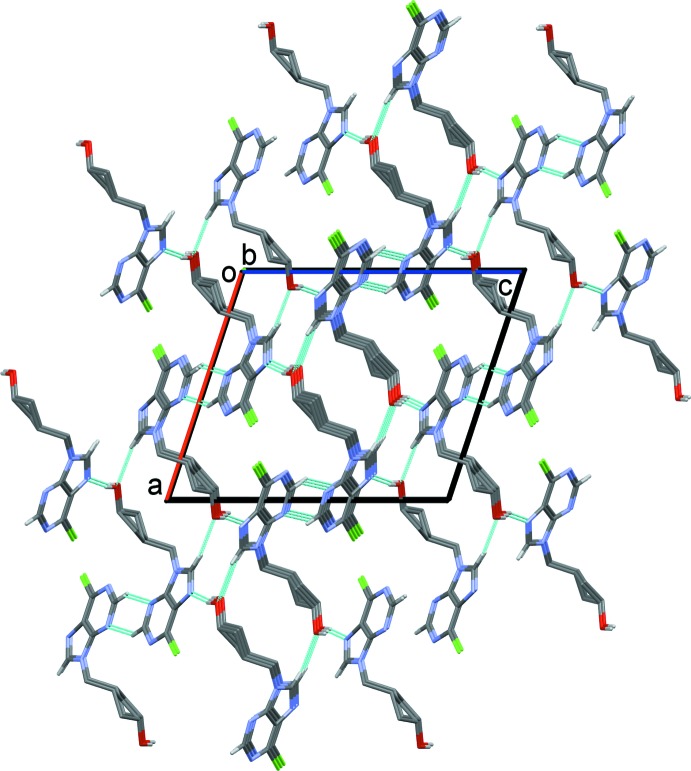
Crystal packing of compound (IV), viewed along the *b* axis. The various hydrogen bonds (Table 3 ▸) are shown as dashed lines. For clarity, only the H atoms involved in these inter­actions have been included.

In the crystal of (IV), mol­ecules are linked by O—H⋯N hydrogen bonds (Table 3 ▸), forming chains along direction [101]. The chains are linked by C—H⋯O and C—H⋯N hydrogen bonds, and C—H⋯π (Table 4 ▸) and offset π–π inter­actions, forming a supra­molecular framework (see Fig. 6 ▸). [Details of the offset π–π inter­actions: *CgB*⋯*CgB* ^vi^ = 3.534 (1) Å, *CgB* is the centroid of the purine ring system, α = 0.02 (10) Å, β = 17.8°, inter­planar distance = 3.364 (1) Å, offset = 1.08 Å, symmetry code (vi) −*x* + 1, −*y* + 1, −*z*. ]

## Database survey   

A search of the Cambridge Structural Database (CSD, Version 5.40, update February 2019; Groom *et al.*, 2016 ▸) found two related structure, *viz*. 6-chloro-9-(3-hy­droxy­methyl-3-hy­droxy­cyclo­but­yl)purine (CSD refcode SOGROV; Boumchita *et al.*, 1991 ▸)) and *cis*-1-bromo­methyl-3-(6-chloro-9*H*-purin-yl)cyclo­butanol (ZUMHAQ; Gharbaoui *et al.*, 1995 ▸). The coordinates are not available for either structure.

## Synthesis and crystallization   

**Synthesis of compounds (I) and (III):** Potassium carbonate (12.0 mmol) was added to a solution of (3-oxo­cyclo­but­yl)methyl benzoate (10.0 mmol) in methanol (20 ml) and stirred for 1 h at room temperature. Saturated sodium bicarbonate (10.0 ml) was added and stirring continued for an additional 15 min. The solvent was evaporated under vacuum, followed by purification by flash column chromatography with ethyl acetate, resulting in 3-(hy­droxy­meth­yl)cyclo­butan-1-one in 70% yield.

3-(Hy­droxy­meth­yl)cyclo­butan-1-one (1 mmol) was dissolved in 10 ml of dry di­chloro­methane and cooled to 195 K. Hunig’s base (3.2 mmol) was added, followed by tri­fluoro­methane­sulfonic anhydride (1 mmol) and the mixture was stirred for 10 min, cooled to 273 K and stirred to obtain the qualitative conversion to (3-oxo­cyclo­but­yl)methyl tri­fluoro­methane­sulfonate.

The (3-oxo­cyclo­but­yl)methyl tri­fluoro­methane­sulfonate (5.61 mmol) was added to a mixture containing 6-chloro-7*H*-purine (5.61 mmol), potassium hydroxide (5.61 mmol), tris­[2-(2-meth­oxy­eth­oxy)eth­yl]amine (0.28 mmol), magnesium sulfate (2 g) and anhydrous aceto­nitrile (100 ml), which was then heated to 333 K for 5 h and cooled to room temperature. The product was purified using 5% methanol and 5% tri­methyl­amine in chloro­form, which yielded two UV-active compounds.

The two UV-active compounds were separated using flash column chromatography with ethyl acetate, giving 51% of the N-9 alkyl­ated derivative; 3-[(6-chloro-9*H*-purin-9-yl)meth­yl]cyclo­butan-1-one (III) and 37% of the N-7 alkyl­ated deriv­ative 3-[(6-chloro-7*H*-purin-7-yl)meth­yl]cyclo­butan-1-one (I).

**Synthesis of 3-[(6-chloro-8,9-di­hydro-7** ***H*** **-purin-7-yl)meth­yl]cyclo­butan-1-ol (II):** 3-[(6-Chloro-7*H*-purin-7-yl)meth­yl]cyclo­butan-1-one (I) (0.21 mmol) in di­chloro­methane (10 ml) was cooled to 195 K and lithium tri-*tert*-but­oxy­aluminum hydride was added. The mixture was cooled to room temperature and sodium borohydride (0.32 mmol) was added and the resulting mixture allowed to stir overnight. Methanol (2 ml) was added and the mixture allowed to stir overnight to convert the over-reduced 3-[(6-chloro-7*H*-purin-7-yl]meth­yl)cyclo­butanone(I) to 3-[(6-chloro-8,9-di­hydro-7*H*-purin-7-yl)meth­yl]cyclo­butan-1-ol (II).

**Synthesis of** ***cis*** **-3-[(6-chloro-9** ***H*** **-purin-9-yl)meth­yl]cyclo­butan-1-ol (IV):** 3-[(6-Chloro-9*H*-purin-9-yl)meth­yl]cyclo­butan-1-one (III) (0.21 mmol) was added to diethyl ether and cooled to 195 K and lithium tri-*tert*-but­oxy­aluminum hydride (0.32 mmol) was added. The reaction was allowed to warm to room temperature and left to stir overnight, which provided qu­anti­tative conversion to *cis*-3-[(6-chloro-9*H*-purin-9-yl)meth­yl]cyclo­butan-1-ol (IV). Crystallization was achieved through evaporation over three days with tetra­hydro­furan as the solvent.

Pale-yellow plate-like crystals of (I), suitable for X-ray diffraction analysis, were obtained by slow evaporation of a solution in di­chloro­methane and heptane. Colourless plate-like crystals of (II), were obtained by slow evaporation of a solution in methanol, di­chloro­methane and diethyl ether (1:1:1, 9 ml). Colorless plate-like crystals of (IV), were obtained by slow evaporation of a solution in methanol (3 ml) .

## Refinement   

Crystal data, data collection and structure refinement details are summarized in Table 5 ▸. For all three compounds, C-bound H atoms were placed in calculated positions and refined as riding: C—H = 0.95–0.99 Å with *U*_iso_(H) = 1.2*U*_eq_(C). For compound (II), the OH and NH H atoms were located in a difference-Fourier map. While the OH H atom was freely refined the NH H atom was refined with a distance restraint: N—H = 0.86 (2) Å. For compound (IV), the OH H atom was located in a difference-Fourier map and freely refined. In compound (II), atoms C6′ and C4′ are positionally disordered and were split giving a refined occupancy ratio for C6′:C6′*B* and C4′:C4′*B* of 0.858 (4):0.142 (4). For the final refinement of compound (II) three most disagreeable reflections (3
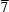
1, 3
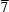
2, 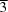
70) were omitted, and for the final refinement of compound (IV) four most disagreeable reflections (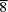
58, 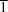
66, 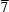
57, 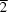
67) were omitted.

## Supplementary Material

Crystal structure: contains datablock(s) global, I, II, IV. DOI: 10.1107/S2056989019004432/zp2033sup1.cif

Structure factors: contains datablock(s) I. DOI: 10.1107/S2056989019004432/zp2033Isup2.hkl

Structure factors: contains datablock(s) II. DOI: 10.1107/S2056989019004432/zp2033IIsup3.hkl

Supporting information file. DOI: 10.1107/S2056989019004432/zp2033IIsup5.cml

CCDC references: 1907200, 1907199, 1907198

Additional supporting information:  crystallographic information; 3D view; checkCIF report

## Figures and Tables

**Table 1:** Hydrogen-bond geometry (Å, °) for (I)

*D*—H⋯*A*	*D*—H	H⋯*A*	*D*⋯*A*	*D*—H⋯*A*
C6′—H6′*B*⋯Cl1	0.99	2.66	3.407 (2)	132

**Table 2:** Hydrogen-bond geometry (Å, °) for (II) *Cg*1 is the centroid of the N1/C2/N3/C4/C5/C6 ring.

*D*—H⋯*A*	*D*—H	H⋯*A*	*D*⋯*A*	*D*—H⋯*A*
C6′—H6′2⋯Cl1	0.99	2.64	3.390 (3)	132
N9—H9⋯N3^i^	0.83 (2)	2.14 (2)	2.952 (2)	166 (2)
O1′—H1′⋯N1^ii^	0.84 (3)	2.09 (3)	2.909 (2)	164 (3)
C4′—H4′⋯*Cg*1^iii^	0.99	2.87	3.857 (3)	170

Symmetry codes: (i) 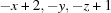; (ii) 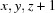; (iii) 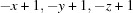.

**Table 3:** Hydrogen-bond geometry (Å, °) for (IV) *Cg*1 is the centroid of the N1/C2/N3/C4/C5/C6 ring.

*D*—H⋯*A*	*D*—H	H⋯*A*	*D*⋯*A*	*D*—H⋯*A*
O1′—H1′⋯N7^i^	0.84	2.03	2.853 (3)	168
C8—H8⋯O1′^ii^	0.95	2.27	3.148 (2)	153
C2—H2⋯N3^iii^	0.95	2.48	3.311 (3)	146
C2′—H2′⋯*Cg*1^iv^	0.99	2.84	3.628 (2)	136

Symmetry codes: (i) 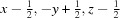; (ii) 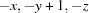; (iii) 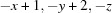; (iv) 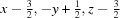.

**Table 4:** Geometric parameters (Å, °) about atom C8 for compounds (I), (II) and (IV)

Bond/angle	(I)	(II)	(IV)
C8—N7	1.381 (2)	1.471 (3)	1.362 (3)
C8—N9	1.301 (2)	1.455 (3)	1.321 (3)
N7—C8—N9	114.95 (15)	103.41 (15)	114.28 (18)

**Table 5:** Experimental details

	(I)	(II)	(IV)
Crystal data			
Chemical formula	C_10_H_9_ClN_4_O	C_10_H_13_ClN_4_O	C_10_H_11_ClN_4_O
*M*_r_	236.66	240.69	238.68
Crystal system, space group	Monoclinic, *P*2_1_/*c*	Triclinic, *P* 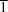	Monoclinic, *P*2_1_/*n*
Temperature (K)	110	110	110
*a*, *b*, *c* (Å)	11.9736 (5), 6.8854 (4), 12.2746 (5)	6.1101 (4), 8.6075 (5), 11.0083 (7)	12.7276 (8), 5.9725 (4), 14.819 (1)
α, β, γ (°)	90, 92.938 (4), 90	68.957 (6), 83.799 (5), 87.189 (5)	90, 108.250 (3), 90
*V* (Å^3^)	1010.63 (8)	537.15 (6)	1069.81 (12)
*Z*	4	2	4
Radiation type	Mo *K*α	Cu *K*α	Cu *K*α
μ (mm^−1^)	0.36	3.03	3.04
Crystal size (mm)	0.43 × 0.21 × 0.04	0.44 × 0.30 × 0.12	0.50 × 0.21 × 0.07

Data collection			
Diffractometer	Bruker APEXII CCD	Bruker–Nonius Kappa CCD	Bruker APEXII CCD
Absorption correction	Multi-scan (*CrysAlis PRO*; Rigaku OD, 2018 ▸)	Multi-scan (*CrysAlis PRO*; Rigaku OD, 2018 ▸)	Numerical (*CrysAlis PRO*; Rigaku OD, 2018 ▸)
*T*_min_, *T*_max_	0.661, 1.000	0.771, 1.000	0.043, 0.741
No. of measured, independent and observed [*I* > 2σ(*I*)] reflections	35539, 2508, 2217	9261, 1793, 1681	7078, 1732, 1569
*R*_int_	0.046	0.028	0.045
(sin θ/λ)_max_ (Å^−1^)	0.667	0.592	0.587

Refinement			
*R*[*F* ^2^ > 2σ(*F* ^2^)], *wR*(*F* ^2^), *S*	0.043, 0.111, 1.07	0.037, 0.098, 1.07	0.038, 0.103, 0.94
No. of reflections	2508	1793	1732
No. of parameters	145	160	146
No. of restraints	0	1	0
H-atom treatment	H-atom parameters constrained	H atoms treated by a mixture of independent and constrained refinement	H-atom parameters constrained
Δρ_max_, Δρ_min_ (e Å^−3^)	0.60, −0.28	0.71, −0.27	0.35, −0.31

Computer programs: *CrysAlis PRO* (Rigaku OD, 2018 ▸), *SHELXS* (Sheldrick, 2008 ▸), *SHELXL* (Sheldrick, 2015 ▸), *PLATON* (Spek, 2009 ▸) and *Mercury* (Macrae *et al.*, 2008 ▸), *OLEX2* (Dolomanov *et al.*, 2009 ▸), *PLATON* (Spek, 2009 ▸) and *publCIF* (Westrip, 2010 ▸).

## supplementary crystallographic information

### 3-[(6-Chloro-7H-purin-7-yl)methyl]cyclobutan-1-one (I) Crystal data

**Table:**

C_10_H_9_ClN_4_O	*F*(000) = 488
*M**_r_* = 236.66	*D*_x_ = 1.555 Mg m^−^^3^
Monoclinic, *P*2_1_/*c*	Mo *K*α radiation, λ = 0.71073 Å
*a* = 11.9736 (5) Å	Cell parameters from 9903 reflections
*b* = 6.8854 (4) Å	θ = 3.3–32.6°
*c* = 12.2746 (5) Å	µ = 0.36 mm^−^^1^
β = 92.938 (4)°	*T* = 110 K
*V* = 1010.63 (8) Å^3^	Plate, pale_yellow
*Z* = 4	0.43 × 0.21 × 0.04 mm

### 3-[(6-Chloro-7H-purin-7-yl)methyl]cyclobutan-1-one (I) Data collection

**Table:**

Bruker APEXII CCD diffractometer	2508 independent reflections
Radiation source: sealed X-ray tube	2217 reflections with *I* > 2σ(*I*)
Graphite monochromator	*R*_int_ = 0.046
ω scans	θ_max_ = 28.3°, θ_min_ = 3.3°
Absorption correction: multi-scan (CrysAlisPro; Rigaku OD, 2018)	*h* = −15→15
*T*_min_ = 0.661, *T*_max_ = 1.000	*k* = −9→9
35539 measured reflections	*l* = −16→16

### 3-[(6-Chloro-7H-purin-7-yl)methyl]cyclobutan-1-one (I) Refinement

**Table:**

Refinement on *F*^2^	Primary atom site location: structure-invariant direct methods
Least-squares matrix: full	Secondary atom site location: difference Fourier map
*R*[*F*^2^ > 2σ(*F*^2^)] = 0.043	Hydrogen site location: inferred from neighbouring sites
*wR*(*F*^2^) = 0.111	H-atom parameters constrained
*S* = 1.07	*w* = 1/[σ^2^(*F*_o_^2^) + (0.0508*P*)^2^ + 0.9477*P*] where *P* = (*F*_o_^2^ + 2*F*_c_^2^)/3
2508 reflections	(Δ/σ)_max_ < 0.001
145 parameters	Δρ_max_ = 0.60 e Å^−^^3^
0 restraints	Δρ_min_ = −0.28 e Å^−^^3^

### 3-[(6-Chloro-7H-purin-7-yl)methyl]cyclobutan-1-one (I) Special details

**Table:**

Geometry. All esds (except the esd in the dihedral angle between two l.s. planes) are estimated using the full covariance matrix. The cell esds are taken into account individually in the estimation of esds in distances, angles and torsion angles; correlations between esds in cell parameters are only used when they are defined by crystal symmetry. An approximate (isotropic) treatment of cell esds is used for estimating esds involving l.s. planes.

### 3-[(6-Chloro-7H-purin-7-yl)methyl]cyclobutan-1-one (I) Fractional atomic coordinates and isotropic or equivalent isotropic displacement parameters (Å^2^)

**Table:**

	*x*	*y*	*z*	*U*_iso_*/*U*_eq_	
Cl1	0.70136 (4)	0.21385 (7)	0.52904 (4)	0.02685 (14)	
N1	0.81306 (13)	0.2530 (2)	0.35301 (12)	0.0230 (3)	
N3	0.93051 (12)	0.5196 (2)	0.30500 (11)	0.0219 (3)	
N7	0.84068 (11)	0.6388 (2)	0.56456 (11)	0.0183 (3)	
N9	0.95030 (12)	0.7744 (2)	0.44062 (12)	0.0216 (3)	
O1'	0.43878 (13)	0.6449 (3)	0.76126 (16)	0.0503 (5)	
C2'	0.53242 (16)	0.6753 (3)	0.73657 (17)	0.0303 (4)	
C3'	0.64220 (16)	0.7042 (3)	0.80129 (15)	0.0297 (4)	
H3'A	0.670598	0.585740	0.839075	0.036*	
H3'B	0.642461	0.816481	0.851695	0.036*	
C4'	0.69801 (14)	0.7464 (3)	0.69174 (14)	0.0215 (4)	
H4'	0.720328	0.885805	0.685533	0.026*	
C5'	0.58520 (15)	0.7032 (3)	0.62724 (16)	0.0297 (4)	
H5'A	0.554763	0.814870	0.584483	0.036*	
H5'B	0.586295	0.584132	0.582080	0.036*	
C6'	0.79428 (14)	0.6106 (3)	0.67259 (12)	0.0198 (3)	
H6'A	0.854160	0.632156	0.729957	0.024*	
H6'B	0.768262	0.474799	0.678746	0.024*	
C2	0.88240 (14)	0.3491 (3)	0.28787 (13)	0.0230 (4)	
H2	0.898377	0.285913	0.221649	0.028*	
C4	0.90857 (13)	0.6006 (3)	0.40103 (13)	0.0183 (3)	
C5	0.83895 (13)	0.5123 (2)	0.47653 (12)	0.0176 (3)	
C6	0.79181 (14)	0.3358 (3)	0.44645 (13)	0.0198 (3)	
C8	0.90917 (14)	0.7900 (3)	0.53629 (14)	0.0205 (3)	
H8	0.925073	0.897356	0.583167	0.025*	

### 3-[(6-Chloro-7H-purin-7-yl)methyl]cyclobutan-1-one (I) Atomic displacement parameters (Å^2^)

**Table:**

	*U*^11^	*U*^22^	*U*^33^	*U*^12^	*U*^13^	*U*^23^
Cl1	0.0305 (2)	0.0264 (2)	0.0241 (2)	−0.00833 (17)	0.00535 (16)	0.00170 (17)
N1	0.0231 (7)	0.0246 (8)	0.0213 (7)	0.0002 (6)	0.0002 (6)	−0.0029 (6)
N3	0.0215 (7)	0.0272 (8)	0.0173 (7)	0.0026 (6)	0.0046 (5)	0.0014 (6)
N7	0.0202 (7)	0.0202 (7)	0.0146 (6)	0.0007 (5)	0.0030 (5)	0.0009 (5)
N9	0.0213 (7)	0.0210 (7)	0.0229 (7)	−0.0006 (6)	0.0036 (5)	0.0027 (6)
O1'	0.0302 (8)	0.0585 (12)	0.0639 (11)	−0.0075 (8)	0.0186 (8)	0.0010 (9)
C2'	0.0257 (9)	0.0282 (10)	0.0377 (10)	0.0011 (7)	0.0102 (8)	−0.0006 (8)
C3'	0.0284 (9)	0.0409 (12)	0.0207 (8)	0.0025 (8)	0.0091 (7)	0.0002 (8)
C4'	0.0198 (8)	0.0263 (9)	0.0187 (8)	0.0019 (6)	0.0046 (6)	0.0022 (6)
C5'	0.0215 (8)	0.0425 (12)	0.0251 (9)	0.0037 (8)	0.0000 (7)	−0.0010 (8)
C6'	0.0228 (8)	0.0253 (9)	0.0116 (7)	0.0016 (7)	0.0033 (6)	0.0011 (6)
C2	0.0229 (8)	0.0301 (9)	0.0162 (7)	0.0041 (7)	0.0021 (6)	−0.0038 (7)
C4	0.0177 (7)	0.0204 (8)	0.0168 (7)	0.0022 (6)	0.0003 (6)	0.0037 (6)
C5	0.0179 (7)	0.0213 (8)	0.0135 (7)	0.0035 (6)	0.0004 (6)	0.0011 (6)
C6	0.0195 (7)	0.0223 (8)	0.0175 (7)	0.0007 (6)	0.0015 (6)	0.0039 (6)
C8	0.0205 (7)	0.0176 (8)	0.0232 (8)	0.0009 (6)	−0.0014 (6)	−0.0022 (6)

### 3-[(6-Chloro-7H-purin-7-yl)methyl]cyclobutan-1-one (I) Geometric parameters (Å, º)

**Table:**

Cl1—C6	1.7372 (17)	C3'—H3'A	0.9900
N1—C6	1.317 (2)	C3'—H3'B	0.9900
N1—C2	1.354 (2)	C4'—C6'	1.512 (2)
N3—C2	1.320 (2)	C4'—C5'	1.559 (2)
N3—C4	1.342 (2)	C4'—H4'	1.0000
N7—C8	1.381 (2)	C5'—H5'A	0.9900
N7—C5	1.387 (2)	C5'—H5'B	0.9900
N7—C6'	1.4765 (19)	C6'—H6'A	0.9900
N9—C8	1.301 (2)	C6'—H6'B	0.9900
N9—C4	1.376 (2)	C2—H2	0.9500
O1'—C2'	1.195 (2)	C4—C5	1.415 (2)
C2'—C3'	1.514 (3)	C5—C6	1.382 (2)
C2'—C5'	1.524 (3)	C8—H8	0.9500
C3'—C4'	1.559 (2)		
			
C6—N1—C2	117.04 (16)	C2'—C5'—H5'B	114.0
C2—N3—C4	113.96 (15)	C4'—C5'—H5'B	114.0
C8—N7—C5	105.24 (13)	H5'A—C5'—H5'B	111.2
C8—N7—C6'	125.49 (14)	N7—C6'—C4'	112.54 (14)
C5—N7—C6'	128.74 (15)	N7—C6'—H6'A	109.1
C8—N9—C4	104.09 (14)	C4'—C6'—H6'A	109.1
O1'—C2'—C3'	133.7 (2)	N7—C6'—H6'B	109.1
O1'—C2'—C5'	133.0 (2)	C4'—C6'—H6'B	109.1
C3'—C2'—C5'	93.29 (14)	H6'A—C6'—H6'B	107.8
C2'—C3'—C4'	88.33 (14)	N3—C2—N1	128.03 (16)
C2'—C3'—H3'A	113.9	N3—C2—H2	116.0
C4'—C3'—H3'A	113.9	N1—C2—H2	116.0
C2'—C3'—H3'B	113.9	N3—C4—N9	126.01 (15)
C4'—C3'—H3'B	113.9	N3—C4—C5	123.02 (16)
H3'A—C3'—H3'B	111.1	N9—C4—C5	110.96 (14)
C6'—C4'—C5'	116.77 (16)	C6—C5—N7	138.56 (15)
C6'—C4'—C3'	112.49 (15)	C6—C5—C4	116.68 (15)
C5'—C4'—C3'	90.22 (14)	N7—C5—C4	104.75 (14)
C6'—C4'—H4'	111.9	N1—C6—C5	121.22 (16)
C5'—C4'—H4'	111.9	N1—C6—Cl1	116.95 (14)
C3'—C4'—H4'	111.9	C5—C6—Cl1	121.83 (13)
C2'—C5'—C4'	87.96 (14)	N9—C8—N7	114.95 (15)
C2'—C5'—H5'A	114.0	N9—C8—H8	122.5
C4'—C5'—H5'A	114.0	N7—C8—H8	122.5
			
O1'—C2'—C3'—C4'	−175.7 (3)	C8—N7—C5—C6	179.2 (2)
C5'—C2'—C3'—C4'	3.32 (16)	C6'—N7—C5—C6	7.2 (3)
C2'—C3'—C4'—C6'	−122.53 (16)	C8—N7—C5—C4	0.10 (17)
C2'—C3'—C4'—C5'	−3.24 (16)	C6'—N7—C5—C4	−171.83 (15)
O1'—C2'—C5'—C4'	175.7 (3)	N3—C4—C5—C6	−0.8 (2)
C3'—C2'—C5'—C4'	−3.32 (16)	N9—C4—C5—C6	−179.83 (14)
C6'—C4'—C5'—C2'	118.72 (16)	N3—C4—C5—N7	178.47 (15)
C3'—C4'—C5'—C2'	3.22 (15)	N9—C4—C5—N7	−0.52 (18)
C8—N7—C6'—C4'	76.0 (2)	C2—N1—C6—C5	−0.4 (2)
C5—N7—C6'—C4'	−113.61 (19)	C2—N1—C6—Cl1	179.16 (13)
C5'—C4'—C6'—N7	72.5 (2)	N7—C5—C6—N1	−177.58 (18)
C3'—C4'—C6'—N7	174.86 (15)	C4—C5—C6—N1	1.4 (2)
C4—N3—C2—N1	2.1 (3)	N7—C5—C6—Cl1	2.9 (3)
C6—N1—C2—N3	−1.5 (3)	C4—C5—C6—Cl1	−178.08 (12)
C2—N3—C4—N9	178.09 (16)	C4—N9—C8—N7	−0.70 (19)
C2—N3—C4—C5	−0.8 (2)	C5—N7—C8—N9	0.4 (2)
C8—N9—C4—N3	−178.22 (16)	C6'—N7—C8—N9	172.66 (15)
C8—N9—C4—C5	0.74 (18)		

### 3-[(6-Chloro-7H-purin-7-yl)methyl]cyclobutan-1-one (I) Hydrogen-bond geometry (Å, º)

**Table:**

*D*—H···*A*	*D*—H	H···*A*	*D*···*A*	*D*—H···*A*
C6′—H6′*B*···Cl1	0.99	2.66	3.407 (2)	132
C4′—H4′···Cl1^i^	1.00	2.97	3.7889 (19)	140
C6′—H6′*B*···N1^ii^	0.99	2.68	3.343 (2)	124

Symmetry codes: (i) *x*, *y*+1, *z*; (ii) *x*, −*y*+1/2, *z*+1/2.

### 3-[(6-Chloro-8,9-dihydro-7H-purin-7-yl)methyl]cyclobutan-1-ol (II) Crystal data

**Table:**

C_10_H_13_ClN_4_O	*Z* = 2
*M**_r_* = 240.69	*F*(000) = 252
Triclinic, *P*1	*D*_x_ = 1.488 Mg m^−^^3^
*a* = 6.1101 (4) Å	Cu *K*α radiation, λ = 1.54184 Å
*b* = 8.6075 (5) Å	Cell parameters from 8605 reflections
*c* = 11.0083 (7) Å	θ = 4.3–65.4°
α = 68.957 (6)°	µ = 3.03 mm^−^^1^
β = 83.799 (5)°	*T* = 110 K
γ = 87.189 (5)°	Plate, colorless
*V* = 537.15 (6) Å^3^	0.44 × 0.30 × 0.12 mm

### 3-[(6-Chloro-8,9-dihydro-7H-purin-7-yl)methyl]cyclobutan-1-ol (II) Data collection

**Table:**

Bruker–Nonius Kappa CCD diffractometer	1793 independent reflections
Radiation source: sealed X-ray tube, Enhance (Cu) X-ray Source	1681 reflections with *I* > 2σ(*I*)
Detector resolution: 7.9 pixels mm^-1^	*R*_int_ = 0.028
ω scans	θ_max_ = 66.0°, θ_min_ = 4.3°
Absorption correction: multi-scan (CrysAlisPro; Rigaku OD, 2018)	*h* = −7→5
*T*_min_ = 0.771, *T*_max_ = 1.000	*k* = −10→10
9261 measured reflections	*l* = −13→12

### 3-[(6-Chloro-8,9-dihydro-7H-purin-7-yl)methyl]cyclobutan-1-ol (II) Refinement

**Table:**

Refinement on *F*^2^	Primary atom site location: structure-invariant direct methods
Least-squares matrix: full	Secondary atom site location: difference Fourier map
*R*[*F*^2^ > 2σ(*F*^2^)] = 0.037	Hydrogen site location: mixed
*wR*(*F*^2^) = 0.098	H atoms treated by a mixture of independent and constrained refinement
*S* = 1.07	*w* = 1/[σ^2^(*F*_o_^2^) + (0.0479*P*)^2^ + 0.5464*P*] where *P* = (*F*_o_^2^ + 2*F*_c_^2^)/3
1793 reflections	(Δ/σ)_max_ < 0.001
160 parameters	Δρ_max_ = 0.71 e Å^−^^3^
1 restraint	Δρ_min_ = −0.27 e Å^−^^3^

### 3-[(6-Chloro-8,9-dihydro-7H-purin-7-yl)methyl]cyclobutan-1-ol (II) Special details

**Table:**

Geometry. All esds (except the esd in the dihedral angle between two l.s. planes) are estimated using the full covariance matrix. The cell esds are taken into account individually in the estimation of esds in distances, angles and torsion angles; correlations between esds in cell parameters are only used when they are defined by crystal symmetry. An approximate (isotropic) treatment of cell esds is used for estimating esds involving l.s. planes.

### 3-[(6-Chloro-8,9-dihydro-7H-purin-7-yl)methyl]cyclobutan-1-ol (II) Fractional atomic coordinates and isotropic or equivalent isotropic displacement parameters (Å^2^)

**Table:**

	*x*	*y*	*z*	*U*_iso_*/*U*_eq_	Occ. (<1)
Cl1	0.16834 (8)	0.41808 (6)	0.40624 (5)	0.02598 (19)	
N1	0.4817 (3)	0.2850 (2)	0.28999 (16)	0.0208 (4)	
C2	0.6669 (3)	0.2012 (3)	0.28642 (19)	0.0208 (4)	
H2	0.719070	0.193795	0.204161	0.025*	
N3	0.7920 (3)	0.1241 (2)	0.38609 (16)	0.0201 (4)	
C4	0.7122 (3)	0.1387 (2)	0.49898 (19)	0.0181 (4)	
C5	0.5139 (3)	0.2254 (2)	0.51675 (19)	0.0175 (4)	
C6	0.4064 (3)	0.2984 (2)	0.40720 (19)	0.0185 (4)	
N7	0.4843 (3)	0.2163 (2)	0.64573 (16)	0.0195 (4)	
C8	0.6671 (3)	0.1184 (3)	0.71453 (19)	0.0217 (4)	
H8A	0.750275	0.184768	0.750830	0.026*	
H8B	0.612778	0.016417	0.786389	0.026*	
N9	0.8027 (3)	0.0776 (2)	0.61286 (16)	0.0212 (4)	
H9	0.916 (3)	0.018 (3)	0.627 (2)	0.021 (6)*	
O1'	0.1576 (3)	0.2578 (2)	1.12373 (14)	0.0278 (4)	
H1'	0.256 (5)	0.284 (4)	1.161 (3)	0.045 (8)*	
C2'	0.2169 (4)	0.3455 (3)	0.9900 (2)	0.0273 (5)	
H2'	0.230429	0.466833	0.973632	0.033*	
C3'	0.4091 (4)	0.2873 (3)	0.9160 (2)	0.0346 (6)	
H3'1	0.446403	0.167641	0.956402	0.041*	
H3'2	0.541933	0.356932	0.895749	0.041*	
C5'	0.0629 (4)	0.3203 (3)	0.8985 (2)	0.0277 (5)	
H5'1	−0.044286	0.412524	0.866879	0.033*	
H5'2	−0.011259	0.211215	0.933326	0.033*	
C4'	0.2642 (4)	0.3281 (3)	0.7998 (2)	0.0240 (6)	0.858 (4)
H4'	0.288618	0.445876	0.739504	0.029*	0.858 (4)
C6'	0.2716 (4)	0.2157 (3)	0.7213 (2)	0.0229 (6)	0.858 (4)
H6'1	0.239112	0.100518	0.781565	0.027*	0.858 (4)
H6'2	0.155181	0.251106	0.660680	0.027*	0.858 (4)
C4'B	0.246 (3)	0.216 (2)	0.8417 (15)	0.0240 (6)	0.142 (4)
H4'B	0.228733	0.092243	0.870206	0.029*	0.142 (4)
C6'B	0.332 (3)	0.308 (2)	0.7043 (15)	0.0229 (6)	0.142 (4)
H6'3	0.206224	0.345077	0.650824	0.027*	0.142 (4)
H6'4	0.406521	0.409522	0.701360	0.027*	0.142 (4)

### 3-[(6-Chloro-8,9-dihydro-7H-purin-7-yl)methyl]cyclobutan-1-ol (II) Atomic displacement parameters (Å^2^)

**Table:**

	*U*^11^	*U*^22^	*U*^33^	*U*^12^	*U*^13^	*U*^23^
Cl1	0.0222 (3)	0.0321 (3)	0.0240 (3)	0.0101 (2)	−0.0049 (2)	−0.0110 (2)
N1	0.0227 (9)	0.0239 (9)	0.0162 (8)	0.0000 (7)	−0.0018 (7)	−0.0078 (7)
C2	0.0227 (11)	0.0255 (10)	0.0158 (10)	−0.0004 (8)	0.0012 (8)	−0.0104 (8)
N3	0.0198 (9)	0.0244 (9)	0.0173 (8)	0.0013 (7)	0.0010 (7)	−0.0100 (7)
C4	0.0171 (10)	0.0192 (10)	0.0186 (10)	−0.0008 (8)	−0.0003 (8)	−0.0080 (8)
C5	0.0181 (10)	0.0193 (9)	0.0166 (10)	−0.0025 (8)	0.0006 (8)	−0.0087 (8)
C6	0.0168 (10)	0.0204 (10)	0.0192 (10)	0.0014 (8)	−0.0004 (8)	−0.0087 (8)
N7	0.0173 (9)	0.0277 (9)	0.0155 (8)	0.0057 (7)	−0.0014 (7)	−0.0109 (7)
C8	0.0212 (11)	0.0293 (11)	0.0176 (10)	0.0059 (8)	−0.0036 (8)	−0.0121 (8)
N9	0.0181 (9)	0.0294 (10)	0.0181 (9)	0.0094 (7)	−0.0038 (7)	−0.0114 (7)
O1'	0.0275 (9)	0.0394 (9)	0.0177 (7)	0.0028 (7)	−0.0043 (6)	−0.0115 (7)
C2'	0.0286 (12)	0.0371 (12)	0.0179 (10)	−0.0017 (9)	−0.0008 (9)	−0.0119 (9)
C3'	0.0257 (12)	0.0522 (15)	0.0340 (13)	0.0000 (11)	−0.0030 (10)	−0.0253 (12)
C5'	0.0250 (11)	0.0400 (13)	0.0215 (11)	0.0119 (9)	−0.0052 (9)	−0.0157 (10)
C4'	0.0258 (13)	0.0263 (14)	0.0205 (12)	0.0035 (11)	0.0002 (10)	−0.0104 (11)
C6'	0.0164 (12)	0.0341 (16)	0.0216 (12)	0.0011 (11)	0.0000 (10)	−0.0147 (11)
C4'B	0.0258 (13)	0.0263 (14)	0.0205 (12)	0.0035 (11)	0.0002 (10)	−0.0104 (11)
C6'B	0.0164 (12)	0.0341 (16)	0.0216 (12)	0.0011 (11)	0.0000 (10)	−0.0147 (11)

### 3-[(6-Chloro-8,9-dihydro-7H-purin-7-yl)methyl]cyclobutan-1-ol (II) Geometric parameters (Å, º)

**Table:**

Cl1—C6	1.739 (2)	C2'—C3'	1.524 (3)
N1—C2	1.317 (3)	C2'—C5'	1.527 (3)
N1—C6	1.365 (3)	C2'—H2'	1.0000
C2—N3	1.355 (3)	C3'—C4'	1.561 (3)
C2—H2	0.9500	C3'—C4'B	1.626 (16)
N3—C4	1.332 (3)	C3'—H3'1	0.9900
C4—N9	1.342 (3)	C3'—H3'2	0.9900
C4—C5	1.424 (3)	C5'—C4'	1.537 (3)
C5—C6	1.366 (3)	C5'—C4'B	1.616 (15)
C5—N7	1.386 (2)	C5'—H5'1	0.9900
N7—C6'B	1.440 (15)	C5'—H5'2	0.9900
N7—C6'	1.465 (3)	C4'—C6'	1.508 (3)
N7—C8	1.471 (3)	C4'—H4'	1.0000
C8—N9	1.455 (3)	C6'—H6'1	0.9900
C8—H8A	0.9900	C6'—H6'2	0.9900
C8—H8B	0.9900	C4'B—C6'B	1.48 (2)
N9—H9	0.833 (17)	C4'B—H4'B	1.0000
O1'—C2'	1.407 (3)	C6'B—H6'3	0.9900
O1'—H1'	0.84 (3)	C6'B—H6'4	0.9900
			
C2—N1—C6	116.73 (17)	C2'—C3'—H3'1	114.0
N1—C2—N3	127.76 (18)	C4'—C3'—H3'1	114.0
N1—C2—H2	116.1	C2'—C3'—H3'2	114.0
N3—C2—H2	116.1	C4'—C3'—H3'2	114.0
C4—N3—C2	113.74 (17)	H3'1—C3'—H3'2	111.2
N3—C4—N9	126.70 (19)	C2'—C5'—C4'	88.64 (17)
N3—C4—C5	124.39 (18)	C2'—C5'—C4'B	92.8 (6)
N9—C4—C5	108.91 (17)	C2'—C5'—H5'1	113.9
C6—C5—N7	136.29 (19)	C4'—C5'—H5'1	113.9
C6—C5—C4	115.33 (18)	C2'—C5'—H5'2	113.9
N7—C5—C4	108.35 (17)	C4'—C5'—H5'2	113.9
N1—C6—C5	122.04 (18)	H5'1—C5'—H5'2	111.1
N1—C6—Cl1	115.38 (15)	C6'—C4'—C5'	118.5 (2)
C5—C6—Cl1	122.56 (15)	C6'—C4'—C3'	120.1 (2)
C5—N7—C6'B	128.7 (6)	C5'—C4'—C3'	87.40 (17)
C5—N7—C6'	125.55 (17)	C6'—C4'—H4'	109.7
C5—N7—C8	108.55 (16)	C5'—C4'—H4'	109.7
C6'B—N7—C8	121.7 (6)	C3'—C4'—H4'	109.7
C6'—N7—C8	118.33 (17)	N7—C6'—C4'	113.3 (2)
N9—C8—N7	103.41 (15)	N7—C6'—H6'1	108.9
N9—C8—H8A	111.1	C4'—C6'—H6'1	108.9
N7—C8—H8A	111.1	N7—C6'—H6'2	108.9
N9—C8—H8B	111.1	C4'—C6'—H6'2	108.9
N7—C8—H8B	111.1	H6'1—C6'—H6'2	107.7
H8A—C8—H8B	109.0	C6'B—C4'B—C5'	112.7 (13)
C4—N9—C8	110.76 (17)	C6'B—C4'B—C3'	99.0 (12)
C4—N9—H9	125.9 (17)	C5'—C4'B—C3'	82.6 (7)
C8—N9—H9	123.2 (17)	C6'B—C4'B—H4'B	118.5
C2'—O1'—H1'	103 (2)	C5'—C4'B—H4'B	118.5
O1'—C2'—C3'	121.1 (2)	C3'—C4'B—H4'B	118.5
O1'—C2'—C5'	114.52 (18)	N7—C6'B—C4'B	115.0 (14)
C3'—C2'—C5'	89.12 (16)	N7—C6'B—H6'3	108.5
O1'—C2'—H2'	110.1	C4'B—C6'B—H6'3	108.5
C3'—C2'—H2'	110.1	N7—C6'B—H6'4	108.5
C5'—C2'—H2'	110.1	C4'B—C6'B—H6'4	108.5
C2'—C3'—C4'	87.87 (18)	H6'3—C6'B—H6'4	107.5
C2'—C3'—C4'B	92.5 (5)		
			
C6—N1—C2—N3	0.8 (3)	N7—C8—N9—C4	−1.3 (2)
N1—C2—N3—C4	−0.3 (3)	O1'—C2'—C3'—C4'	138.2 (2)
C2—N3—C4—N9	−178.94 (19)	C5'—C2'—C3'—C4'	19.78 (18)
C2—N3—C4—C5	0.4 (3)	O1'—C2'—C3'—C4'B	105.1 (6)
N3—C4—C5—C6	−1.1 (3)	C5'—C2'—C3'—C4'B	−13.3 (6)
N9—C4—C5—C6	178.36 (17)	O1'—C2'—C5'—C4'	−144.2 (2)
N3—C4—C5—N7	−179.35 (17)	C3'—C2'—C5'—C4'	−20.09 (19)
N9—C4—C5—N7	0.1 (2)	O1'—C2'—C5'—C4'B	−110.7 (6)
C2—N1—C6—C5	−1.6 (3)	C3'—C2'—C5'—C4'B	13.4 (6)
C2—N1—C6—Cl1	177.14 (14)	C2'—C5'—C4'—C6'	142.7 (2)
N7—C5—C6—N1	179.3 (2)	C2'—C5'—C4'—C3'	19.62 (18)
C4—C5—C6—N1	1.6 (3)	C2'—C3'—C4'—C6'	−141.4 (2)
N7—C5—C6—Cl1	0.7 (3)	C2'—C3'—C4'—C5'	−19.66 (18)
C4—C5—C6—Cl1	−176.96 (14)	C5—N7—C6'—C4'	−135.6 (2)
C6—C5—N7—C6'B	−10.1 (10)	C8—N7—C6'—C4'	78.4 (3)
C4—C5—N7—C6'B	167.6 (10)	C5'—C4'—C6'—N7	−171.4 (2)
C6—C5—N7—C6'	32.6 (4)	C3'—C4'—C6'—N7	−66.6 (3)
C4—C5—N7—C6'	−149.7 (2)	C2'—C5'—C4'B—C6'B	−109.4 (12)
C6—C5—N7—C8	−178.7 (2)	C2'—C5'—C4'B—C3'	−12.7 (6)
C4—C5—N7—C8	−0.9 (2)	C2'—C3'—C4'B—C6'B	124.6 (10)
C5—N7—C8—N9	1.3 (2)	C2'—C3'—C4'B—C5'	12.7 (6)
C6'B—N7—C8—N9	−168.2 (9)	C5—N7—C6'B—C4'B	146.2 (9)
C6'—N7—C8—N9	152.69 (18)	C8—N7—C6'B—C4'B	−46.6 (16)
N3—C4—N9—C8	−179.79 (18)	C5'—C4'B—C6'B—N7	−175.7 (10)
C5—C4—N9—C8	0.8 (2)	C3'—C4'B—C6'B—N7	98.5 (13)

### 3-[(6-Chloro-8,9-dihydro-7H-purin-7-yl)methyl]cyclobutan-1-ol (II) Hydrogen-bond geometry (Å, º)

Cg1 is the centroid of ring N1/C2/N3/C4/C5/C6.

**Table:**

*D*—H···*A*	*D*—H	H···*A*	*D*···*A*	*D*—H···*A*
C6′—H6′2···Cl1	0.99	2.64	3.390 (3)	132
N9—H9···N3^i^	0.83 (2)	2.14 (2)	2.952 (2)	166 (2)
O1′—H1′···N1^ii^	0.84 (3)	2.09 (3)	2.909 (2)	164 (3)
C4′—H4′···*Cg*1^iii^	0.99	2.87	3.857 (3)	170

Symmetry codes: (i) −*x*+2, −*y*, −*z*+1; (ii) *x*, *y*, *z*+1; (iii) −*x*+1, −*y*+1, −*z*+1.

### 3-[(6-Chloro-9H-purin-9-yl)methyl]cyclobutan-1-ol (IV) Crystal data

**Table:**

C_10_H_11_ClN_4_O	*F*(000) = 496
*M**_r_* = 238.68	*D*_x_ = 1.482 Mg m^−^^3^
Monoclinic, *P*2_1_/*n*	Cu *K*α radiation, λ = 1.54178 Å
*a* = 12.7276 (8) Å	Cell parameters from 4964 reflections
*b* = 5.9725 (4) Å	θ = 4.0–64.9°
*c* = 14.819 (1) Å	µ = 3.04 mm^−^^1^
β = 108.250 (3)°	*T* = 110 K
*V* = 1069.81 (12) Å^3^	Plate, colourless
*Z* = 4	0.50 × 0.21 × 0.07 mm

### 3-[(6-Chloro-9H-purin-9-yl)methyl]cyclobutan-1-ol (IV) Data collection

**Table:**

Bruker APEXII CCD diffractometer	1732 independent reflections
Radiation source: sealed X-ray tube	1569 reflections with *I* > 2σ(*I*)
Graphite monochromator	*R*_int_ = 0.045
Detector resolution: 7.9 pixels mm^-1^	θ_max_ = 64.9°, θ_min_ = 4.0°
ω scans	*h* = −14→14
Absorption correction: numerical (CrysAlisPro; Rigaku OD, 2018)	*k* = −4→7
*T*_min_ = 0.043, *T*_max_ = 0.741	*l* = −17→17
7078 measured reflections	

### 3-[(6-Chloro-9H-purin-9-yl)methyl]cyclobutan-1-ol (IV) Refinement

**Table:**

Refinement on *F*^2^	Primary atom site location: structure-invariant direct methods
Least-squares matrix: full	Secondary atom site location: difference Fourier map
*R*[*F*^2^ > 2σ(*F*^2^)] = 0.038	Hydrogen site location: inferred from neighbouring sites
*wR*(*F*^2^) = 0.103	H-atom parameters constrained
*S* = 0.94	*w* = 1/[σ^2^(*F*_o_^2^) + (0.0599*P*)^2^ + 1.4189*P*] where *P* = (*F*_o_^2^ + 2*F*_c_^2^)/3
1732 reflections	(Δ/σ)_max_ < 0.001
146 parameters	Δρ_max_ = 0.35 e Å^−^^3^
0 restraints	Δρ_min_ = −0.31 e Å^−^^3^

### 3-[(6-Chloro-9H-purin-9-yl)methyl]cyclobutan-1-ol (IV) Special details

**Table:**

Geometry. All esds (except the esd in the dihedral angle between two l.s. planes) are estimated using the full covariance matrix. The cell esds are taken into account individually in the estimation of esds in distances, angles and torsion angles; correlations between esds in cell parameters are only used when they are defined by crystal symmetry. An approximate (isotropic) treatment of cell esds is used for estimating esds involving l.s. planes.

### 3-[(6-Chloro-9H-purin-9-yl)methyl]cyclobutan-1-ol (IV) Fractional atomic coordinates and isotropic or equivalent isotropic displacement parameters (Å^2^)

**Table:**

	*x*	*y*	*z*	*U*_iso_*/*U*_eq_	
Cl1	0.65978 (4)	0.22748 (10)	0.21719 (4)	0.0258 (2)	
O1'	−0.06577 (11)	0.5696 (3)	−0.19248 (10)	0.0233 (4)	
H1'	−0.067187	0.456986	−0.226798	0.035*	
N3	0.44729 (14)	0.7995 (3)	0.06539 (12)	0.0218 (4)	
N1	0.61729 (14)	0.5895 (3)	0.11346 (12)	0.0196 (4)	
N7	0.39848 (13)	0.3289 (3)	0.19580 (12)	0.0199 (4)	
N9	0.30402 (13)	0.6280 (3)	0.11873 (11)	0.0186 (4)	
C4	0.40817 (16)	0.6422 (4)	0.11001 (13)	0.0178 (5)	
C5	0.46564 (16)	0.4569 (4)	0.15808 (13)	0.0168 (5)	
C3'	0.12604 (16)	0.5194 (4)	−0.07173 (14)	0.0201 (5)	
H3'A	0.090406	0.436047	−0.031431	0.024*	
H3'B	0.179230	0.424010	−0.090614	0.024*	
C6	0.57424 (16)	0.4399 (4)	0.15774 (13)	0.0180 (5)	
C8	0.30351 (16)	0.4385 (4)	0.16970 (14)	0.0192 (5)	
H8	0.240264	0.389301	0.185165	0.023*	
C2	0.55210 (18)	0.7598 (4)	0.07007 (15)	0.0223 (5)	
H2	0.584609	0.865250	0.038801	0.027*	
C2'	0.04431 (16)	0.6477 (4)	−0.15487 (14)	0.0177 (5)	
H2'	0.077386	0.673588	−0.206946	0.021*	
C4'	0.17237 (18)	0.7495 (4)	−0.03173 (15)	0.0216 (5)	
H4'	0.231950	0.796287	−0.058547	0.026*	
C6'	0.21115 (17)	0.7800 (4)	0.07552 (15)	0.0208 (5)	
H6'A	0.234806	0.936928	0.091463	0.025*	
H6'B	0.149539	0.747512	0.100968	0.025*	
C5'	0.05979 (17)	0.8537 (4)	−0.09087 (15)	0.0217 (5)	
H5'A	0.005170	0.866225	−0.056036	0.026*	
H5'B	0.066593	0.996160	−0.122717	0.026*	

### 3-[(6-Chloro-9H-purin-9-yl)methyl]cyclobutan-1-ol (IV) Atomic displacement parameters (Å^2^)

**Table:**

	*U*^11^	*U*^22^	*U*^33^	*U*^12^	*U*^13^	*U*^23^
Cl1	0.0169 (3)	0.0313 (4)	0.0291 (3)	0.0052 (2)	0.0069 (2)	0.0101 (2)
O1'	0.0144 (7)	0.0311 (10)	0.0231 (8)	−0.0006 (6)	0.0037 (6)	−0.0095 (7)
N3	0.0217 (9)	0.0229 (11)	0.0184 (9)	−0.0037 (8)	0.0027 (7)	0.0013 (8)
N1	0.0194 (9)	0.0206 (10)	0.0183 (8)	−0.0042 (8)	0.0051 (7)	−0.0005 (8)
N7	0.0155 (9)	0.0260 (11)	0.0177 (8)	−0.0011 (8)	0.0045 (7)	0.0024 (8)
N9	0.0142 (8)	0.0232 (11)	0.0160 (8)	0.0021 (8)	0.0013 (7)	−0.0017 (8)
C4	0.0169 (10)	0.0206 (12)	0.0129 (9)	−0.0030 (9)	0.0004 (8)	−0.0016 (8)
C5	0.0156 (9)	0.0207 (12)	0.0121 (9)	−0.0022 (8)	0.0014 (7)	−0.0014 (8)
C3'	0.0188 (10)	0.0231 (12)	0.0177 (10)	0.0034 (9)	0.0045 (8)	−0.0013 (9)
C6	0.0160 (10)	0.0219 (12)	0.0134 (9)	−0.0010 (9)	0.0006 (8)	−0.0002 (9)
C8	0.0159 (10)	0.0234 (13)	0.0177 (9)	−0.0013 (9)	0.0045 (8)	0.0003 (9)
C2	0.0230 (11)	0.0232 (13)	0.0193 (10)	−0.0064 (9)	0.0047 (9)	0.0015 (9)
C2'	0.0134 (9)	0.0220 (12)	0.0165 (10)	0.0005 (9)	0.0031 (8)	−0.0007 (9)
C4'	0.0186 (11)	0.0249 (13)	0.0194 (11)	0.0010 (9)	0.0035 (9)	0.0009 (9)
C6'	0.0193 (11)	0.0209 (12)	0.0194 (10)	0.0043 (9)	0.0021 (8)	0.0000 (9)
C5'	0.0206 (10)	0.0223 (13)	0.0185 (10)	0.0032 (9)	0.0011 (8)	0.0001 (9)

### 3-[(6-Chloro-9H-purin-9-yl)methyl]cyclobutan-1-ol (IV) Geometric parameters (Å, º)

**Table:**

Cl1—C6	1.723 (2)	C3'—C2'	1.544 (3)
O1'—C2'	1.415 (2)	C3'—H3'A	0.9900
O1'—H1'	0.8400	C3'—H3'B	0.9900
N3—C4	1.332 (3)	C8—H8	0.9500
N3—C2	1.335 (3)	C2—H2	0.9500
N1—C6	1.325 (3)	C2'—C5'	1.528 (3)
N1—C2	1.342 (3)	C2'—H2'	1.0000
N7—C8	1.321 (3)	C4'—C6'	1.520 (3)
N7—C5	1.388 (3)	C4'—C5'	1.556 (3)
N9—C8	1.362 (3)	C4'—H4'	1.0000
N9—C4	1.374 (3)	C6'—H6'A	0.9900
N9—C6'	1.469 (3)	C6'—H6'B	0.9900
C4—C5	1.393 (3)	C5'—H5'A	0.9900
C5—C6	1.388 (3)	C5'—H5'B	0.9900
C3'—C4'	1.539 (3)		
			
C2'—O1'—H1'	109.5	N3—C2—H2	116.0
C4—N3—C2	111.81 (19)	N1—C2—H2	116.0
C6—N1—C2	117.32 (17)	O1'—C2'—C5'	115.42 (17)
C8—N7—C5	103.39 (18)	O1'—C2'—C3'	119.16 (18)
C8—N9—C4	106.00 (17)	C5'—C2'—C3'	88.88 (16)
C8—N9—C6'	127.78 (17)	O1'—C2'—H2'	110.6
C4—N9—C6'	126.10 (18)	C5'—C2'—H2'	110.6
N3—C4—N9	127.7 (2)	C3'—C2'—H2'	110.6
N3—C4—C5	126.60 (19)	C6'—C4'—C3'	118.02 (19)
N9—C4—C5	105.69 (18)	C6'—C4'—C5'	118.75 (18)
C6—C5—N7	134.5 (2)	C3'—C4'—C5'	88.05 (16)
C6—C5—C4	114.87 (19)	C6'—C4'—H4'	110.1
N7—C5—C4	110.64 (17)	C3'—C4'—H4'	110.1
C4'—C3'—C2'	86.87 (17)	C5'—C4'—H4'	110.1
C4'—C3'—H3'A	114.2	N9—C6'—C4'	109.58 (17)
C2'—C3'—H3'A	114.2	N9—C6'—H6'A	109.8
C4'—C3'—H3'B	114.2	C4'—C6'—H6'A	109.8
C2'—C3'—H3'B	114.2	N9—C6'—H6'B	109.8
H3'A—C3'—H3'B	111.3	C4'—C6'—H6'B	109.8
N1—C6—C5	121.3 (2)	H6'A—C6'—H6'B	108.2
N1—C6—Cl1	117.18 (15)	C2'—C5'—C4'	86.82 (16)
C5—C6—Cl1	121.52 (16)	C2'—C5'—H5'A	114.2
N7—C8—N9	114.28 (18)	C4'—C5'—H5'A	114.2
N7—C8—H8	122.9	C2'—C5'—H5'B	114.2
N9—C8—H8	122.9	C4'—C5'—H5'B	114.2
N3—C2—N1	128.1 (2)	H5'A—C5'—H5'B	111.3

### 3-[(6-Chloro-9H-purin-9-yl)methyl]cyclobutan-1-ol (IV) Hydrogen-bond geometry (Å, º)

Cg1 is the centroid of ring N1/C2/N3/C4/C5/C6.

**Table:**

*D*—H···*A*	*D*—H	H···*A*	*D*···*A*	*D*—H···*A*
O1′—H1′···N7^i^	0.84	2.03	2.853 (3)	168
C8—H8···O1′^ii^	0.95	2.27	3.148 (2)	153
C2—H2···N3^iii^	0.95	2.48	3.311 (3)	146
C2′—H2′···*Cg*1^iv^	0.99	2.84	3.628 (2)	136

Symmetry codes: (i) *x*−1/2, −*y*+1/2, *z*−1/2; (ii) −*x*, −*y*+1, −*z*; (iii) −*x*+1, −*y*+2, −*z*; (iv) *x*−3/2, −*y*+1/2, *z*−3/2.
